# Supplementary figures and images for: Four Markers Useful for the Distinction of Intrauterine Growth Restriction in Sheep
Source: Animals (Basel). 2023 Oct 24;13(21):3305. doi: 10.3390/ani13213305 (PMC10648371; doi:10.3390/ani13213305)

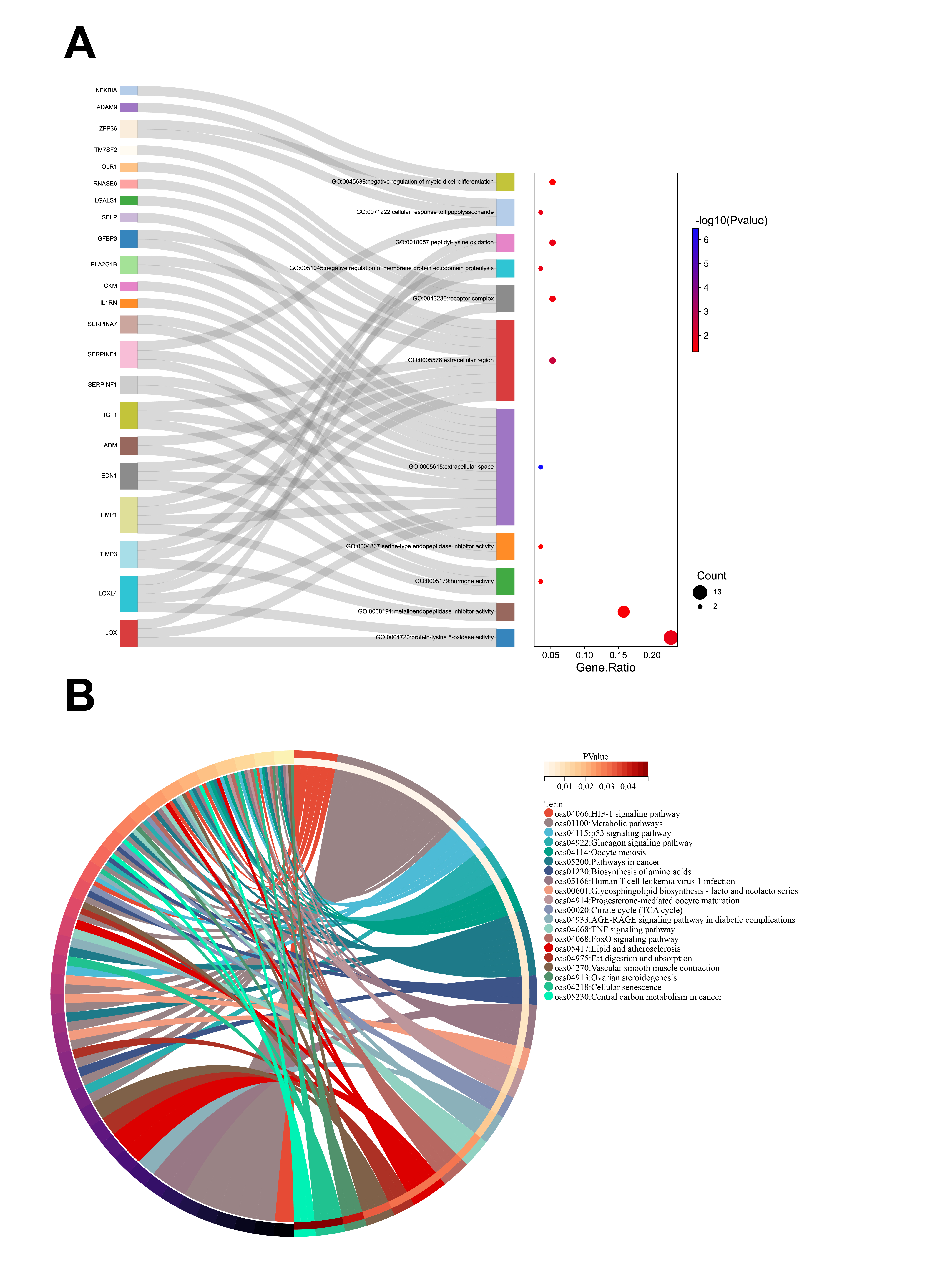

Supplement: Supplementary file 1 [file animals-13-03305-s001.zip › Figure S1.jpg]

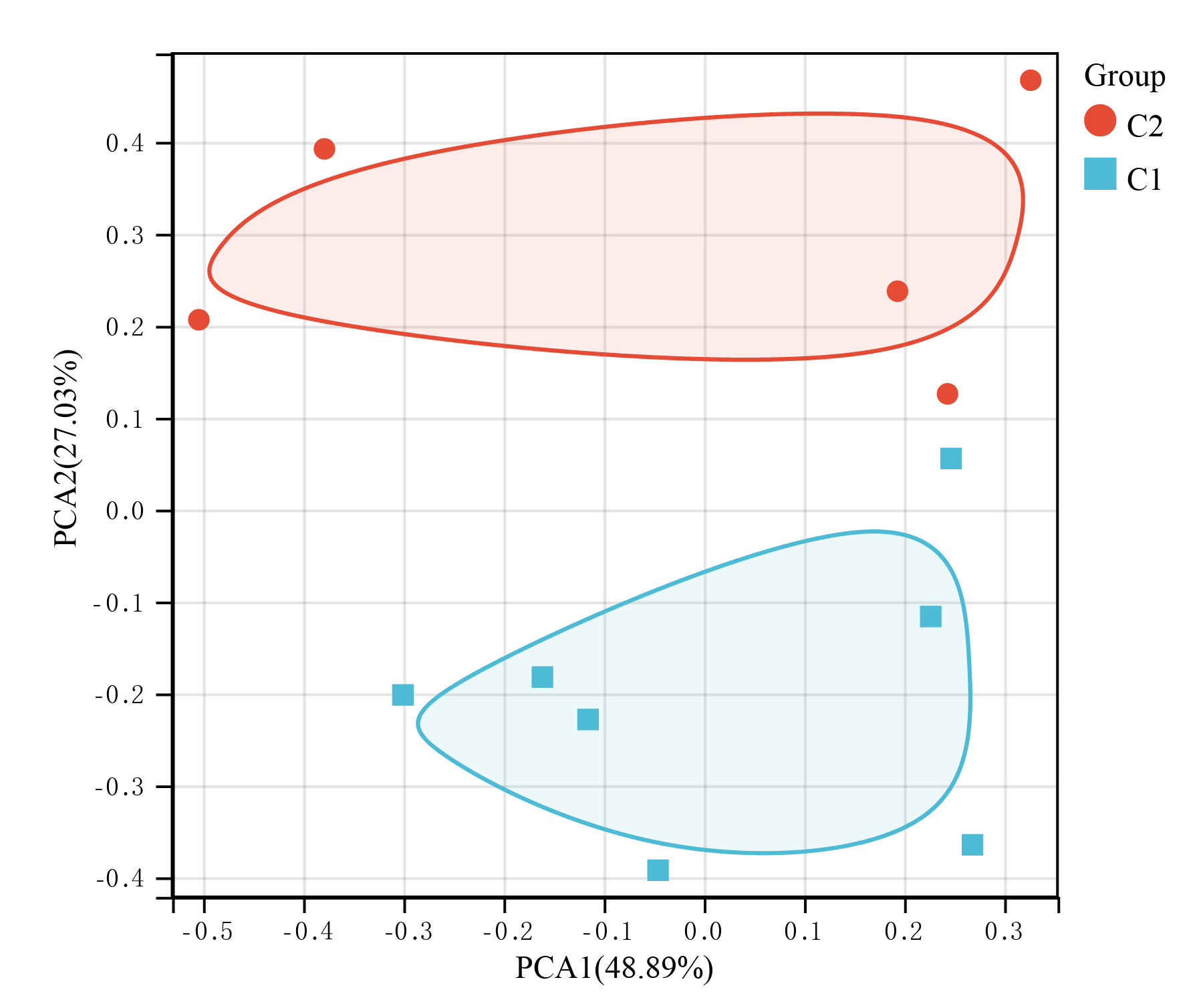

Supplement: Supplementary file 1 [file animals-13-03305-s001.zip › Figure S2.jpg]

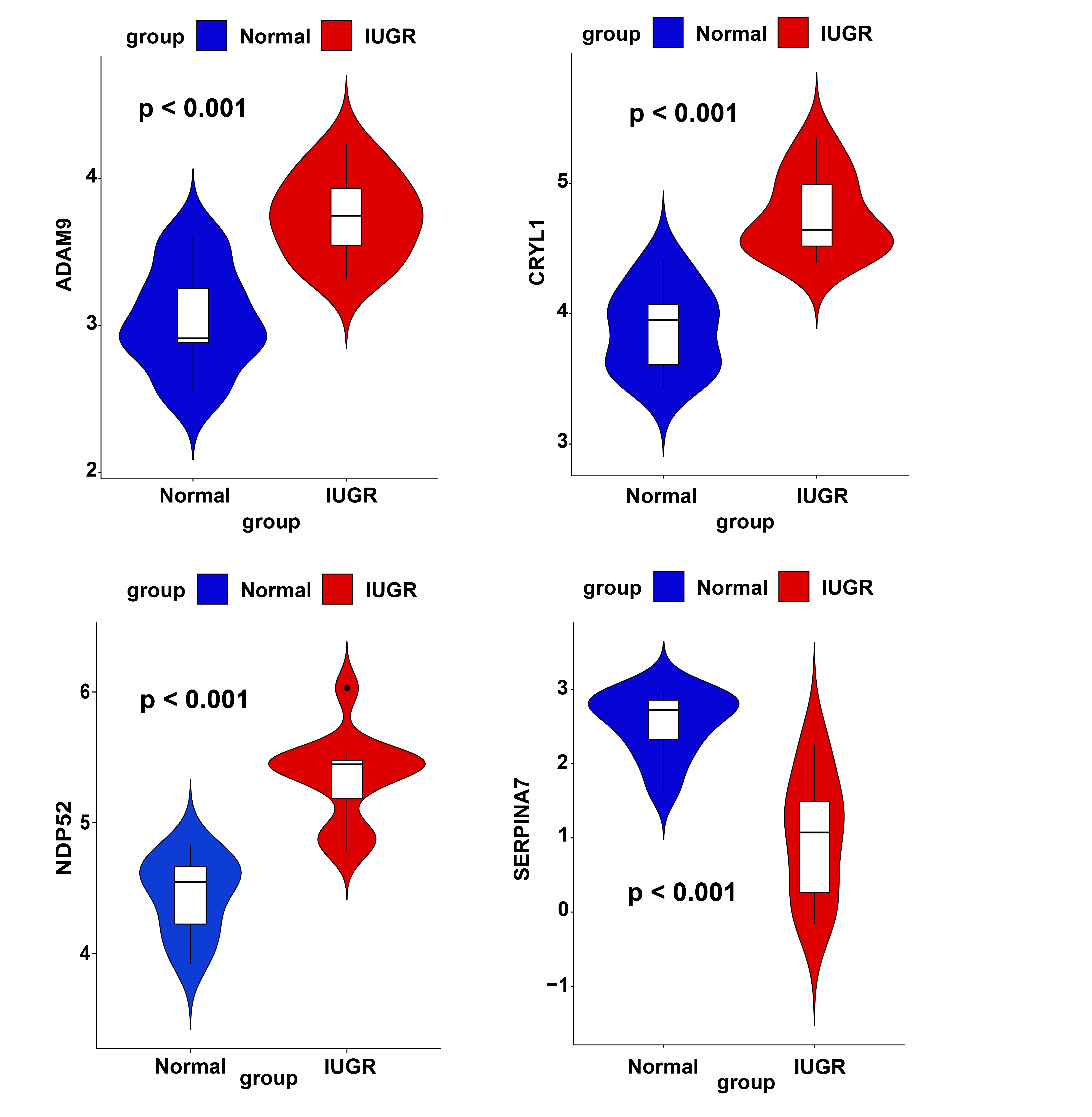

Supplement: Supplementary file 1 [file animals-13-03305-s001.zip › Figure S3.jpg]

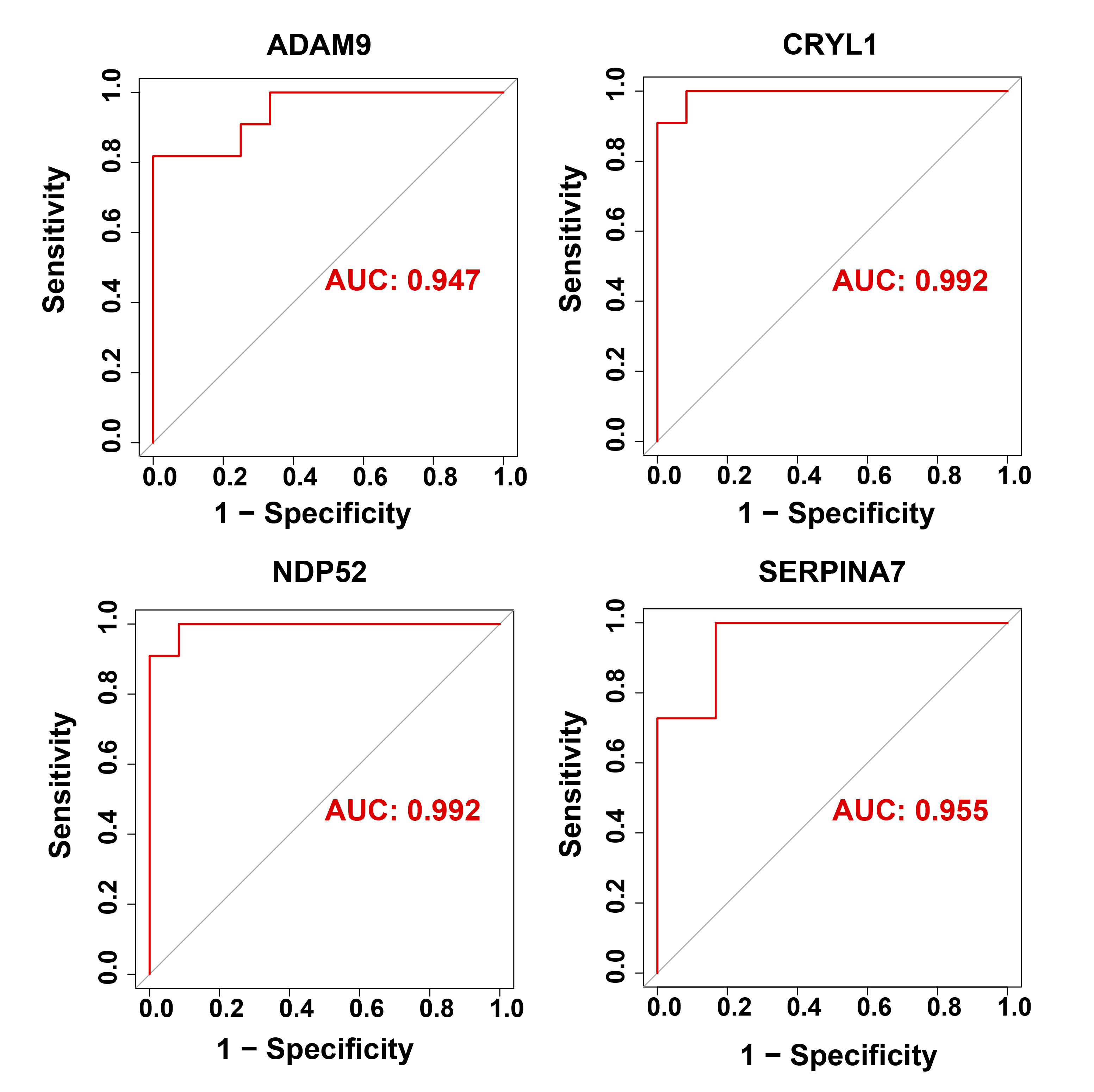

Supplement: Supplementary file 1 [file animals-13-03305-s001.zip › Figure S4.jpg]

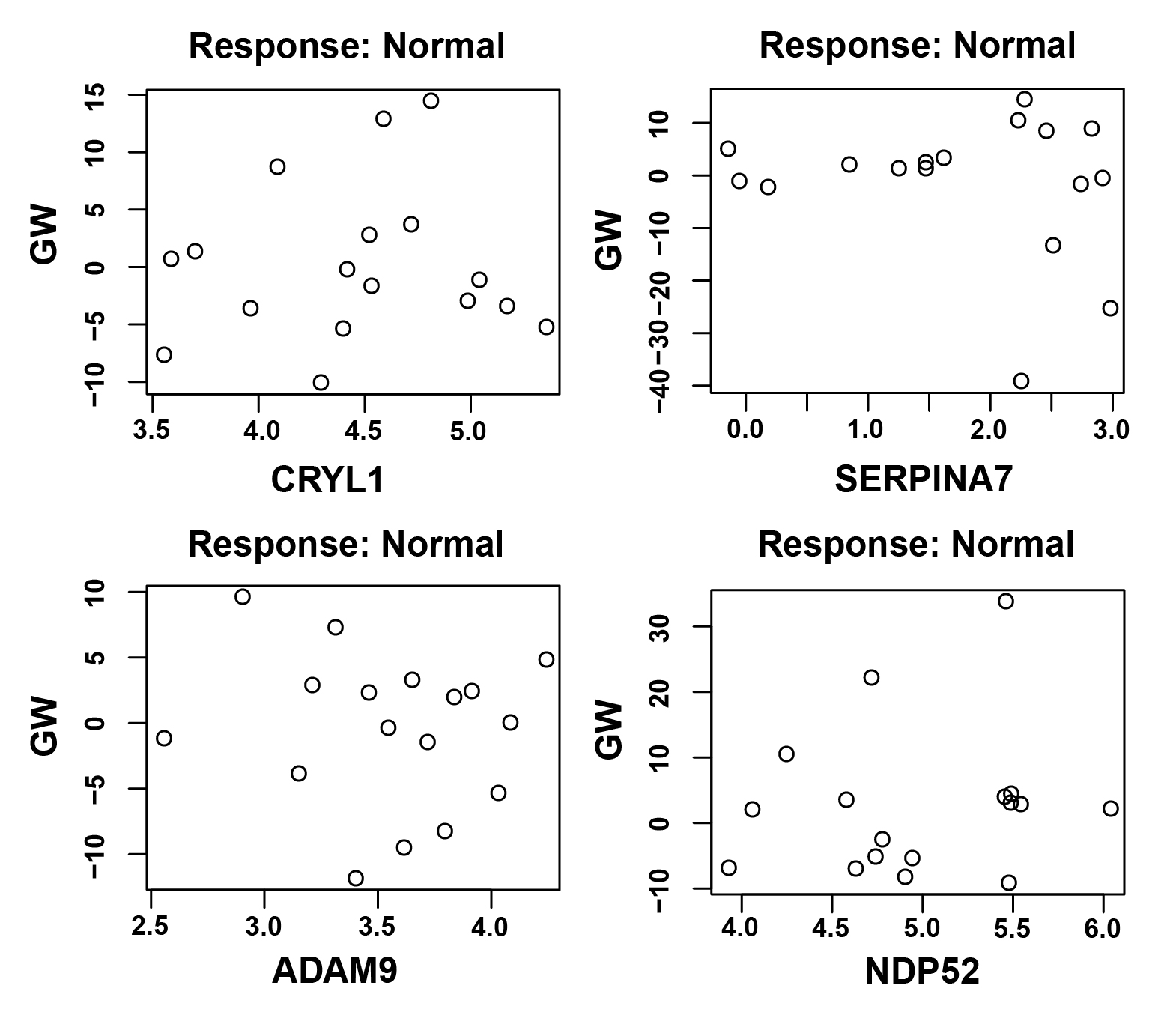

Supplement: Supplementary file 1 [file animals-13-03305-s001.zip › Figure S5.jpg]
